# Waterborne Outbreak of Gastroenteritis: Effects on Sick Leaves and Cost of Lost Workdays

**DOI:** 10.1371/journal.pone.0033307

**Published:** 2012-03-19

**Authors:** Jaana I. Halonen, Mika Kivimäki, Tuula Oksanen, Pekka Virtanen, Mikko J. Virtanen, Jaana Pentti, Jussi Vahtera

**Affiliations:** 1 Finnish Institute of Occupational Health, Helsinki, Finland; 2 Department of Epidemiology and Public Health, University College of London, London, United Kingdom; 3 Tampere School of Health Sciences, University of Tampere, Tampere, Finland; 4 Epidemiologic Surveillance and Response Unit, National Institute for Health and Welfare, Helsinki, Finland; 5 Department of Public Health, University of Turku, and Turku University Hospital, Turku, Finland; Aga Khan University, Pakistan

## Abstract

**Background:**

In 2007, part of a drinking water distribution system was accidentally contaminated with waste water effluent causing a gastroenteritis outbreak in a Finnish town.

We examined the acute and cumulative effects of this incidence on sick leaves among public sector employees residing in the clean and contaminated areas, and the additional costs of lost workdays due to the incidence.

**Methods:**

Daily information on sick leaves of 1789 Finnish Public Sector Study participants was obtained from employers' registers. Global Positioning System-coordinates were used for linking participants to the clean and contaminated areas. Prevalence ratios (PR) for weekly sickness absences were calculated using binomial regression analysis. Calculations for the costs were based on prior studies.

**Results:**

Among those living in the contaminated areas, the prevalence of participants on sick leave was 3.54 (95% confidence interval (CI) 2.97–4.22) times higher on the week following the incidence compared to the reference period. Those living and working in the clean area were basically not affected, the corresponding PR for sick leaves was 1.12, 95% CI 0.73–1.73. No cumulative effects on sick leaves were observed among the exposed. The estimated additional costs of lost workdays due to the incidence were 1.8–2.1 million euros.

**Conclusions:**

The prevalence of sickness absences among public sector employees residing in affected areas increased shortly after drinking water distribution system was contaminated, but no long-term effects were observed. The estimated costs of lost workdays were remarkable, thus, the cost-benefits of better monitoring systems for the water distribution systems should be evaluated.

## Introduction

Regardless of the developed systems for drinking water purification and distribution, and the active legislation towards safe water [1], microbial contamination of drinking water and waterborne infections keep occurring also in developed societies [2], [3], [4], [5], [6], [7], [8]. Large populations may become affected if public water systems are contaminated. These incidences may be due to system deficiencies, improper water treatment or contamination of one part of, or the whole, water supply [3], [4], [7]. In most developed countries with public water distribution systems, however, rapid actions for managing the incidences can be taken to limit the harms; clean water can often be provided by temporary arrangements and the distribution systems can be disinfected. Severities of the waterborne outbreaks are often evaluated by calculating excess cases or risk rates for morbidity among the exposed [2], [4], [5]. However, the effects of the waterborne infections on acute and cumulative sickness absences (*Campylobacter*, *Salmonella* and *Giardia* infections may also have long-term health effects [9], [10], [11], [12]) and the related costs of lost workdays have rarely been determined [13].

In late November 2007 part of the municipal drinking water system in a Finnish town Nokia, a municipality participating in the Finnish Public Sector Study, was contaminated with wastewater effluent (including *Campylobacter* sp., Norovirus, *Giardia*, and *Salmonella* sp.) causing an outbreak of gastroenteritis [6]. In this study, we determined how this epidemic affected the sick leaves immediately and in the following year among employees of the Public Sector Study cohort who resided in Nokia. We hypothesized that employees living in the contaminated areas would be more affected than those living in the non-contaminated “clean” areas. We also estimated the additional costs of lost workdays for all branches of industry during the acute phase of the epidemic.

## Methods

### Ethics statement

All the register data obtained from the national registers were based on the legal permissions granted by institutes maintaining these registers, and data were analyzed anonymously. According to Finnish Personal Data Act (523/1999, Chapter 4, Section 14) [14], written consent was not needed for research that uses register data. The Coordinating Ethics Committee of Helsinki and Uusimaa Hospital District has approved the study.

**Figure 1:**
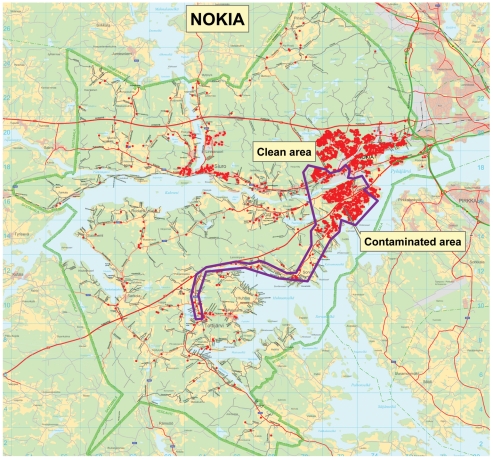
Distribution of cohort participants within areas receiving contaminated and clean drinking water. Dots in the inner pattern represent cohort participants' residences receiving contaminated water.

The Finnish Public Sector Study cohort consists of employees working for 10 municipalities (including Nokia and neighbouring town Tampere) and 21 hospitals (including Tampere University Hospital). All men and women employed by these organizations for more than six months in any year between 1991 and 2005 were eligible for the cohort (n = 151 618). Of this cohort, 1789 members were employed and resided in Nokia during the outbreak (weeks 48/2007–8/2008 referred to as week of outbreak = 0, and weeks after +1, +2, etc. up to week +12) and formed the analytic sample of this study. Because in Finland female-dominated occupations are common in the public sector (nurses, kindergarten teachers, cleaners etc.) a majority of the study population were women. All information about cohort members' job contracts, age, sex and sick leaves was obtained from the employers' registers. For each day we obtained data on all sick leaves (due to own illness), and sick-child leaves (due to illness of a child <10 years), however, diagnoses for the illnesses were not available as they are not recorded by the employers. Sick days (up to 60 days) and sick-child days (allowed absence up to four days at one time) are paid by the employer and, thus, carefully recorded. Residential addresses of the cohort members and Global Positioning System (GPS)-coordinates of these addresses were obtained from Population Register Center. Coordinates of the buildings alongside the contaminated water line were in the National Institute for Health and Welfare. Using these coordinates the cohort members' residences we linked to the buildings to which contaminated drinking water was distributed. The locations of cohort participants' residences in relation to areas where contaminated and clean drinking water were supplied are shown in Figure 1.

The incidence has been described in detail previously [6]. In short, during maintenance work at the Nokia wastewater plant on November 28^th^ 2007 (week 0) a valve connecting wastewater effluent line and drinking water line was accidentally opened, and in some areas inhabitants started to receive contaminated municipal drinking water (referred to as contaminated areas: inner pattern in Figure 1). This area was approximately 18 km^2^ in size (whole town 347 km^2^), and defined based on the addresses and GPS-coordinates of the buildings along the branch of the contaminated distribution system. Two days later, an increase in the number of patients with symptoms of gastroenteritis was observed at the local health care center. An estimated 450 m^3^ of wastewater effluent had been mixed to the drinking water and distributed to the customers, until the water contamination was realized. Recommendation for boiling tap water was issued the same day, clean water distribution organized by municipal officials, the military, and volunteers was started, as well as increased chlorination of the distribution system. After the incident schools and day care centres were closed due to Christmas holiday on weeks +4 and +5. The last restrictions in water use were canceled on February 18^th^ 2008 (week +12).

### Statistical analyses

We calculated the weekly prevalence of study population taking at least one sick day or sick-child day during each week for seven weeks before, during, and 12 weeks after the incidence, separately for those living in the contaminated and clean areas. For contaminated and clean areas cumulative sick days per person year were also calculated for the 52 weeks following the incidence. This was because there is evidence of long-term health effects of *Campylobacter* infections (rheumatological complaints, symptoms of arthritis, inflammatory bowel disease) [9], [10], [15], *Giardia* infections (functional gastrointestinal diseases) [12], and *Salmonella* infections (inflammatory bowel disease) [15].

We calculated the prevalence ratios (PR) and 95% confidence intervals (CI) for weekly sickness absences using log-binomial regression analyses with GENMOD procedure of SAS [16], adjusting for age and sex. The reference period used in the analyses was the mean weekly prevalence of participants taking at least one sick day during the seven weeks prior to the incidence (weeks −7 to −1). The same method was used for the subgroup analyses of the cohort members consisting of those employed by schools and day care centers (n = 361). For them, the data included the location of their residence and workplace, and information of whether these were in the contaminated or clean area. Thus, we could estimate more explicitly the role of clean and contaminated areas in the sickness absences.

The additional cost of lost workdays on weeks 0 to +3 was estimated for the total number of employees residing in Nokia based on the number of additional (compared to reference period) personal and sick-child days per person among the cohort participants. The costs were estimated at 280 and 330 euros per day; the mean estimated costs of sickness absence for the service sector (200–250 €/day), industry (300–350 €/day) [17] and government employees (340–380 €/day) [18] in Finland. Of the total workforce in Nokia in 2007 (n = 10,719), 30% was employed by the government and 32% by industry. These estimations considered the contamination status of the residential area, and were calculated by sex (n of employed women in town = 5020, n of employed men in town = 5699) because the sex distribution of the analytic sample was skewed and women generally have higher rates of sick leaves than men [19].

## Results

The total population in Nokia was 30 016 of which 9538 (32%) lived in the area of contaminated water distribution system. Correspondingly, of the 1789 cohort members 586 (33%) lived in the contaminated area. Of the study population 1419 (79.5%) were women, and the mean age was 45.9 years (standard deviation 9.4). The number of cohort members who worked at schools and day care centers in Nokia was 361, of which 174 lived and worked in the clean area, and 187 lived or worked, or both, in the contaminated area.

**Figure 2:**
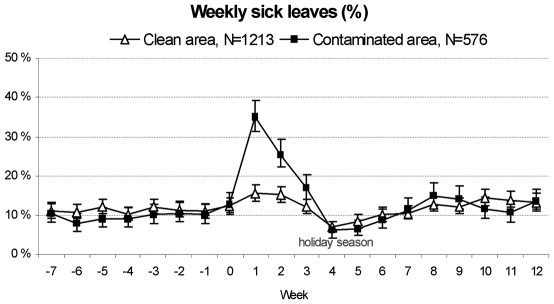
Weekly percentages of participants at sick leave before and after the contamination of water distribution system. Percentages are given by the contamination status of the participants' residence.

**Figure 3:**
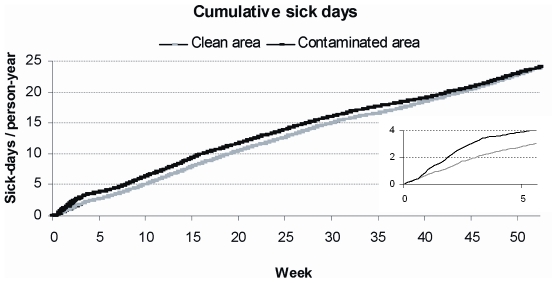
Cumulative sick days for the 52 weeks following the contamination by the contamination status of residence.

Sick leaves increased sharply after the contamination among those who lived in the contaminated area, but not among those living in the clean area for whom the prevalence returned back to the pre-incident level in three weeks (Figure 2). The prevalence of cohort members taking at least one sick day during the week immediately after the contamination (week +1) was 35%, whereas in the week before the contamination the corresponding figure was 10% (week −1). The prevalence of participants at sick-child leave also peaked in the contaminated area on week +1, being 4.3%, whereas the week before (week −1) the level was 1.3% (data not shown). The cumulative number of sick days grew at a faster rate during the first post-incident weeks among those who lived in the contaminated compared to clean area (Figure 3). However, later the curves for cumulative sick days developed similarly for the exposed and non-exposed and by the end of the year the difference had disappeared.

Compared to the reference period, the proportion of participants at sick leave in the contaminated area was the highest on the week following the incidence after which the prevalence ratio decreased gradually (Table 1). A slight increase in the sick leaves was observed also among those living in the clean area with the highest prevalence ratio for week +1. In the subgroup of personnel of schools and day care centers, we found that for those living and working in clean areas, the prevalence ratios for sick leaves remained at the same level after, compared to prior, the contamination (Table 2).

**Table 1:** Prevalence ratios (PR, 95% confidence intervals) for weekly sick leaves among public sector employees after the contamination of drinking water distribution system by contamination status of residence.

	Clean area (n = 1203^a^)			Contaminated area (n = 586^b^)		
Week	PR^c^	95% CI		PR^c^	95% CI	
−7 to −1 (ref)	1			1		
0	1.16	1.02	1.31	1.23	0.99	1.52
+1	1.41	1.23	1.61	3.54	2.97	4.22
+2	1.41	1.23	1.62	2.63	2.18	3.16
+3	1.17	1.01	1.36	1.88	1.52	2.31
+4–12	1.12	1.00	1.26	1.18	0.99	1.40

a Number of participants living in clean area;

b Number of participants living in contaminated area,

c Models adjusted for age and sex.

**Table 2:** Prevalence ratios (PR, 95% confidence intervals) of weekly sick leaves among the personnel of schools and day care centres after the contamination of drinking water distribution system by contamination status of residence and workplace.

	Clean area^a^ (n = 174)			Contaminated area^b^ (n = 187)		
Week	PR^c^	95% CI		PR^c^	95% CI	
−7 to −1 (ref)	1			1		
0	0.90	0.56	1.42	1.21	0.80	1.85
+1	1.12	0.73	1.73	3.43	2.58	4.55
+2	1.10	0.71	1.73	2.89	2.14	3.89
+3	1.03	0.67	1.58	1.36	0.93	2.00
+4–12	1.16	0.88	1.55	1.23	0.96	1.61

a Residence and workplace in the clean area;

b Residence or workplace, or both, in the contaminated area;

c Models adjusted for age and sex.

For the cost estimations we calculated weekly additional sick days (compared to the reference period) in this study population. For the clean and contaminated areas, the sum of additional sick days per person on weeks 0 to +3 was 0.71 for women and 0.42 for men. Thus, the estimated auxiliary cost of lost workdays due to sick leaves among all employees residing in Nokia were 1.00–1.18 and 0.67–0.80 million euros for women and men, respectively. Similarly, the sum of additional sick-child days per person were 0.07 and 0.02, and the estimated cost of lost workdays 0.11–0.13 and 0.03–0.04 million euros for women and men, respectively. These resulted in a total of 1.8–2.1 million euros from lost workdays due to own illness and illness of one's child(ren).

## Discussion

We studied the effect of microbial contamination of drinking water distribution system on sick leaves among public sector employees. Sick- and sick-child leaves were found to increase sharply after the contamination, and this increase was restricted to people either residing or working in the contaminated areas. However, 1-year accumulation of sick days was similar for exposed and unexposed suggesting the effects of the incidence were short-term. The estimated additional costs of lost workdays due to the incidence were 1.8–2.1 million euros.

Contaminations of water distribution systems in the scale of this study are rare, although some have been reported [2], [4], [20]. As far as we are aware, the effects of these incidences on sick leaves have scarcely been studied. We found that those residing in the contaminated area were heavily affected, while those residing and working in the clean areas were unaffected. The peak in the proportion of participants at sick leave was observed during the week after the contamination, which is in agreement with the peak in the detected cases of gastroenteritis related to this incidence [6]. This delay is also in line with other studies on waterborne outbreaks [21], [22].

For the following year, we found no difference in the sick leave accruals between those living in the contaminated and clean areas during the incidence. This suggests the acute epidemic had no residual effects at a population level. Supporting evidence has been reported in regard of occurrence of reactive arthritis in this study area half a year after the incidence [23]. However, prior studies have shown that the effects of gastroenteritis from drinking water contaminated with *Campylobacter* may materialize later -with a delay of up to fifteen years- as increase in arthritis symptoms or inflammatory bowel disease [10], [15], [24], or in case of *Giardia* infection as functional gastrointestinal diseases [12]. Thus, we cannot exclude the possibility that the exposed may still be at increased risk of developing chronic conditions.

We estimated that the excess costs due to sick leaves related to this incidence was 1.8–2.1 million euros, a remarkable part of the total expenditure of the whole incidence that reaches possibly five to six million euros. The other expenses include e.g. the distribution of clean water for the inhabitants, cleaning of the water distribution system, and overtime work of the town and water works employees. As in occupational health in general [25], cost-benefit analyses are needed to estimate whether actions such as online monitoring systems of the water distribution networks would be beneficial in preventing and limiting the magnitude of waterborne outbreaks and all harms related to them. We found that only one study, almost two decades ago, has estimated costs of a waterborne gastroenteritis outbreak. The authors concluded the outbreak affecting 2000–3000 people caused costs of 1.6 million Danish krones (∼215 000 euros in current exchange rate) [13]. However, the algorithm of this estimate was not reported, and because of the long delay between studies the comparison of the costs is difficult. We also found that sick leaves and sick-child leaves were more common among women than men, a finding in line with prior research [19]. Indeed, only a fourth of the costs of sick-child leaves were observed among men, which suggests that female-dominated sectors suffer considerably more from the illnesses of children than male-dominated sectors.

Major strengths of this study are the reliable and comprehensive records on the cohort members' sickness absences and information whether contaminated water was distributed to their residences. Additional information on the locations of workplaces for a sub-population of the cohort enabled us a more detailed examination of the differences in sick leaves between the exposed and non-exposed. One limitation of this study is that we could not determine the portion of the sick leaves that was actually related to the increase in infections of the digestive system because data on diagnoses were not available; however, it is unlikely that the observed short-term increase in the sick leaves is due to illnesses not related to the incidence. Because e.g. salaries and insurance costs vary by industry, we also emphasize that the calculations for the costs of sick leaves depend on the country and employer in question. Finally, due to differences in infrastructure and public services, the generalizability of these findings in less developed regions may be limited.

In summary, we have shown that the prevalence of sickness absences among public sector employees residing in affected areas increased considerably after drinking water distribution system was contaminated. The estimated costs of lost workdays were remarkable, thus, the cost-benefits of better monitoring systems for the water distribution systems should be evaluated.
